# Asymptomatic carriage of *Plasmodium falciparum* by individuals with variant blood groups and haemoglobin genotypes in southern Ghana

**DOI:** 10.1186/s12936-020-03299-1

**Published:** 2020-06-23

**Authors:** Festus K. Acquah, Dickson Donu, Dorcas Bredu, Sophia Eyia-Ampah, Jones A. Amponsah, Joseph Quartey, Evans K. Obboh, Bernice A. Mawuli, Linda E. Amoah

**Affiliations:** 1 Immunology Department, Noguchi Memorial Institute for Medical Research (NMIMR), University of Ghana, Legon, P. O. Box LG 581, Accra, Ghana; 2 West African Centre for Cell Biology of Infectious Pathogens (WACCBIP), University of Ghana, Volta Road, Legon, P.O. Box LG 54, Accra, Ghana; 3 Parasitology Department, Noguchi Memorial Institute for Medical Research (NMIMR), University of Ghana, Legon, P. O. Box LG 581, Accra, Ghana; 4 School of Medical Sciences, University of Cape Coast, PMB, Cape Coast, Ghana

## Abstract

**Background:**

The ABO and the Rhesus blood group systems, as well as various abnormal haemoglobin (Hb) variants (haemoglobinopathies) are known to influence malaria parasite carriage and disease severity in individuals living in malaria endemic areas. This study identified the blood group and Hb variant distribution and *Plasmodium falciparum* infection status of afebrile individuals living in southern Ghana.

**Methods:**

Afebrile participants were recruited from Obom (358) in the Greater Accra Region and Ewim (100) and Simiw (329) in the Central Region of Ghana. Venous blood (1 ml) was collected into EDTA vacutainer tubes. Three 20 μl drops of blood were used for blood group analysis using the tile method. Another 500 μl aliquot was used for the qualitative sickling test using sodium metabisulphite and haemoglobin electrophoresis. Genomic DNA was extracted from 100 μl of whole blood and used in *P. falciparum* species-specific PCR.

**Results:**

The most abundant blood group and abnormal haemoglobin variant in both sites was blood group O + (47.4%) and HbAS (15.8%). A total of 13 (1.7%) of the participants had full haemoglobinopathies (SS, SC and CC), whilst 196 (25.4%) were carriers (AS and AC). Although there was a significantly higher prevalence of sickling positive participants from the Central Region, genotyping identified a similar prevalence of each of the abnormal haemoglobin genes in both sites. Asymptomatic parasite carriage estimated by PCR was 40.9% in the Central Region and 41.8% in the Greater Accra Region.

**Conclusions:**

Asymptomatic carriage of *P. falciparum* parasite in the study population was not associated with any particular blood group variant or haemoglobin genotype.

## Background

Asymptomatic carriage of *Plasmodium falciparum* parasites is a major challenge to malaria control efforts in all malaria endemic countries. Some studies have identified factors including ABO blood type as well as haemoglobinopathies to alter asymptomatic carriage of malaria parasites [1]. In malaria endemic areas, the ABO blood group has been associated with disease severity, whereas blood group O has been shown to offer protection against severe malaria [2] by minimizing the formation of rosettes [3]; blood group A is linked to severe malaria [4].

The haemoglobin variants are structurally abnormal globin proteins formed as a result of mutations in the beta globin subunit of the haemoglobin gene. These mutations are mainly missense mutations causing amino acid substitutions. The most predominant haemoglobin variants identified in sub-Saharan Africa are HbS and HbC [5]. Haemoglobin S (sickle cell trait) is formed as a result of a mutation in the beta globin gene that leads to a replacement of glutamic acid at amino acid residue 6 with valine. Individuals who inherit an S gene from each parent are homozygous for HbS and have sickle cell anaemia (HbSS), whilst the heterogygous AS individuals are referred to as carriers of the sickle cell trait [5]. Studies have shown that individuals who have the sickle cell trait (HbAS) have 50–90% reduction in parasite density [6], most likely due to reduced parasite invasion and growth retardation [7, 8]. More so, children who are AS have been reported to show faster clearance of asymptomatic malaria infections [9]. Haemoglobin C (HbC) results when the glutamic acid residue at position 6 is replaced with a lysine residue [10, 11]. Individuals with HbCC have been found to enhance malaria transmission by harboring highly infectious gametocytes [12]. Children with HbAC have also been found to carry high loads of asexual stage parasites in addition to gametocytes [13].

This study set out to assess the prevalence of people with haemoglobinopathies as well as the distribution of blood group variants and determine their association with asymptomatic carriage of *P. falciparum* parasites.

## Methods

### Ethical considerations

Ethical approval for the study was obtained from the Institutional Review Board of the Noguchi Memorial Institute for Medical Research (#024/14-15 and 089/14-15). Written consent was obtained from all the study participants. Parental consent was sought for participants who were below 18 years old and for those between 12 and 17 years old, Child assent was also obtained.

### Study sites

The study sites were Obom, Ewim and Simiw all located within the Coastal savannah zone of Ghana. Obom is a rural community in the Greater Accra Region. Obom has perennial malaria transmission and has been classified in the past as a high transmission region that presents high asymptomatic parasite prevalence [14]. Ewim is a sub-urban community in the Cape-Coast municipality (CCMA) of the Central Region with low and perennial malaria transmission [14]. Simiw is a rural setting in the Komenda Edina Eguafo Abrem District (KEEA) of the Central Region and is adjacent to the Cape-Coast municipality. Malaria transmission in Simiw is also perennial. Although malaria is perenial in these three communities, it peaks between June and August.

### Study design and sampling

The study was cross-sectional; and it involved 772 afebrile adults and children who were recruited from November and December of 2019. Venous whole blood (1 ml) was collected from each participant into EDTA vacutainer tubes. A drop (5 µl) of blood was used to spot the Urit 12 (Accurex Biomedical Private Limited, India) haemoglobin meter according to manufacturer’s instructions. Three drops of blood were spotted onto a tile for the ABO blood group typing and a fourth drop used for the erythrocyte haemoglobin phenotyping. The remaining blood was separated into plasma and packed blood cell pellets. The cells were stored at 4 °C and used for the haemoglobin electrophoresis and the plasma was stored at − 20 °C for future use. The axillary temperature of each participant was taken with a digital thermometer.

### Case definition

*Asymptomatic:* an individual with PCR detectable *P. falciparum* parasites on the day of sampling without associated malaria symptoms such as fever.

### ABO and RhD blood group typing

The tile method [15] was used with very minor modifications. Briefly, three 20 µl drops of whole blood were spotted onto a white tile and each blood spot was mixed with an equal volume of anti-A, anti-B or anti-D (Fortress Diagnostic Limited, UK.). The presence of agglutination after 5–10 min of rocking the tile denoted the presence of red cell antigen or rhesus (Rh) antigen corresponding to the anti-sera used. Lack of agglutination for both A and B denoted the blood group O.

### Qualitative sickling test

The sodium metabisulphite method [16] was used to determine the presence of HbS in the whole blood sample. Briefly, a 20 µl drop of blood was spotted on to a glass slide. Subsequently, 20 µl of 2% sodium metabisulphite was added and mixed; and a cover slip placed on top of the mixture. It was incubated for an hour; and after that, the slides were read under 10X and confirmed under 40X objective lens. The presence of sickled red blood cell(s) indicated a sickling positive test and a normal biconcave RBCs indicated a negative test.

### Haemoglobin electrophoresis

The cellulose acetate paper method was used [16]. Briefly, 300 µl of the packed blood cells was washed with 2.5 ml of 0.85% normal saline and centrifuged at 3000 rpm for 3 min. The supernatant was discarded, and the cells were washed two more times as done previously. A few drops of distilled water were added to the cells to lyse the erythrocytes. The haemolysate was applied to the wells of a buffered cellulose acetate membrane (pH 8.6) along with control samples containing haemoglobin A, S, and C. The membrane was then put in the electrophoretic tank and run at 200 V for 45 min. Control samples migrations were used to classify the samples into their appropriate genotypes.

### PCR detection of *Plasmodium falciparum*

The 18S rRNA gene of *P. falciparum* was amplified by nested PCR using previously published protocols [17, 18]. A 15 µl primary reaction mixture was prepared using about 30 ng of DNA, 2.5 mM MgCl_2_, 200 nM dNTP mix, 200 nM each of the rPLU5 and rPLU6 primer set and 1 U of OneTaq DNA polymerase (NEB, UK). A 15 µl secondary reaction mixture contained 2 µl of the primary PCR product (template), 133.33 nM each rFal1 and rFal2, forward and reverse primer respectively, in addition to a similar composition of MgCl_2_ and dNTPs and One Taq as the primary reaction mixture. A no template negative control as well as *P. falciparum* positive controls, MRA-102G (3D7) and MRA-155G (HB3) were included in each set of PCR amplifications. PCR products were resolved on a 2% agarose gel (pre-stained with ethidium bromide) and the gel image was captured under UV with a Vilber gel documentation system (Vilber, France).

### Data analysis

Statistical analyses were done using the IBM SPSS Statistics version 20 package. The demographic details of the study participants from the two study sites were compared using the unpaired *t* test. The association of asymptomatic parasite carriage with site as well as the distribution of the blood groups and haemoglobinopathies was determined using the Pearson Chi Squared test. Similarly, the distribution of *P. falciparum* by haemoglobin genotype and phenotype were also compared using the Pearson Chi Squared test.

## Results

Participants from Central Region (Ewim and Simiw) represented 53.5% (413/772) of the total population and the rest were from the Greater Accra Region (Obom). There were more females (74.9%) than males in the Central Region but a comparable number of males and females recruited from the Greater Accra Region. Participants from the Greater Accra Region had a significantly younger population (p < 0.0001) with significantly lower axillary temperature (p = 0.004), but higher haematocrit levels than the participants from the Central Region (Table 1). *Plasmodium falciparum* prevalence was about 40% in both sites (Table 1).

**Table 1:** Demographic characteristics of study participants

	Central	Greater Accra	Total	p value (T test)
Female (%)	74.9	53.4	64.9	<0.0001*
Age (years)				<0.0001
Mean	24.3	18.5	21.6	
SEM	0.9	0.7	0.6	
Minimum	3.0	3.0	3.0	
Maximum	86.0	75.0	86.0	
Temp (^o^C)				= 0.004
Mean	36.4	36.5	36.4	
SEM	0.0	0.0	0.0	
Minimum	33.6	32.2	32.2	
Maximum	37.7	37.5	37.7	
Haematocrit (g/dL)				< 0.0001
Mean	11.1	12.6	11.8	
SEM	0.1	0.1	0.1	
Minimum	6.9	8.0	6.9	
Maximum	17.8	17.8	17.8	
*P. falciparum*				
Positive (%)	40.9	41.8	41.3	= 0.799*

*SEM* standard error of the mean, *temp* temperature

*p value estimated using Pearson Chi square

### Distribution of blood group variants

**Fig. 1:**
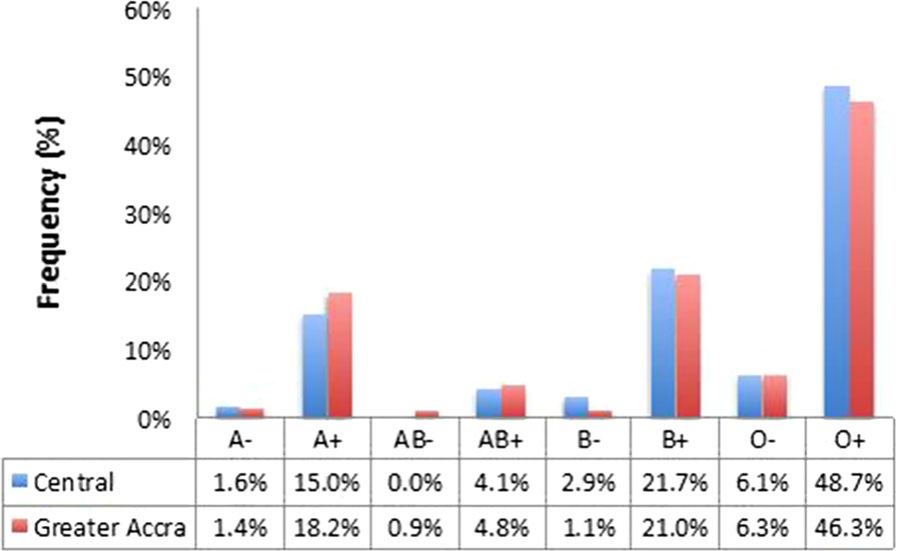
Distribution of blood group variants. The prevalence of each blood group variant amongst the population sampled from each study site presented as a percent of the total population from each site

**Fig. 2:**
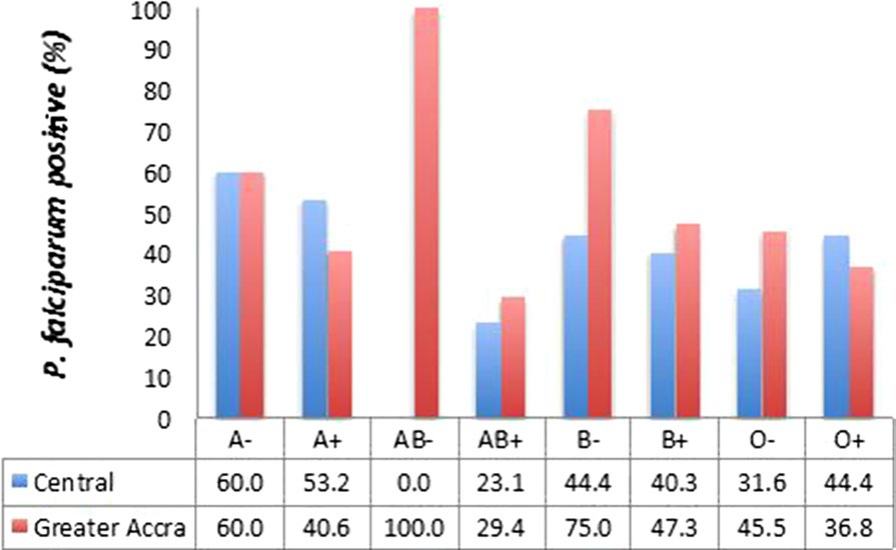
Asymptomatic carriage of *P. falciparum* by the blood group variants. The prevalence of *P. falciparum* parasites carried as asymptomatic infections by study participant belonging to each of the blood group types. The data is presented as a percent of the total number of members within the blood group from each site

The pattern of distribution of the blood group variants was similar (Pearson Chi Square = 6.703, p = 0.460) in both sites; blood group O + and AB− being the most and least prevalent variants respectively (Fig. 1). For this reason, the data for both sites were pooled together for the statistical analyses of asymptomatic *P. falciparum* carriage. Only about 40% of the individuals with blood group O + harbored *P. falciparum* in asymptomatic infections. Asymptomatic malaria parasite carriage in the B + and A + blood groups, which were the second and third most prevalent blood group variants respectively ranged between 40 and 53% (Fig. 2).

### Distribution of haemoglobinopathies

**Fig. 3:**
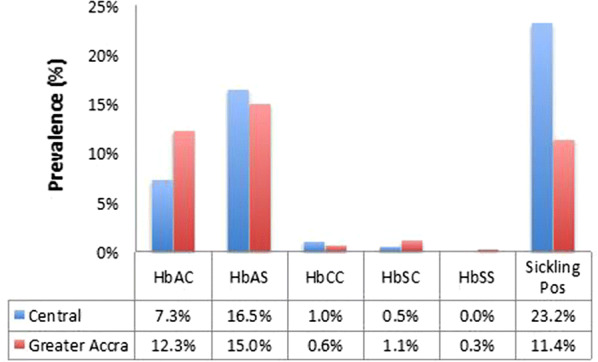
Distribution of abnormal haemoglobin variants. The proportion of the study population that had variant haemoglobin types at each study site presented as a percent of the total population from each site

**Fig. 4:**
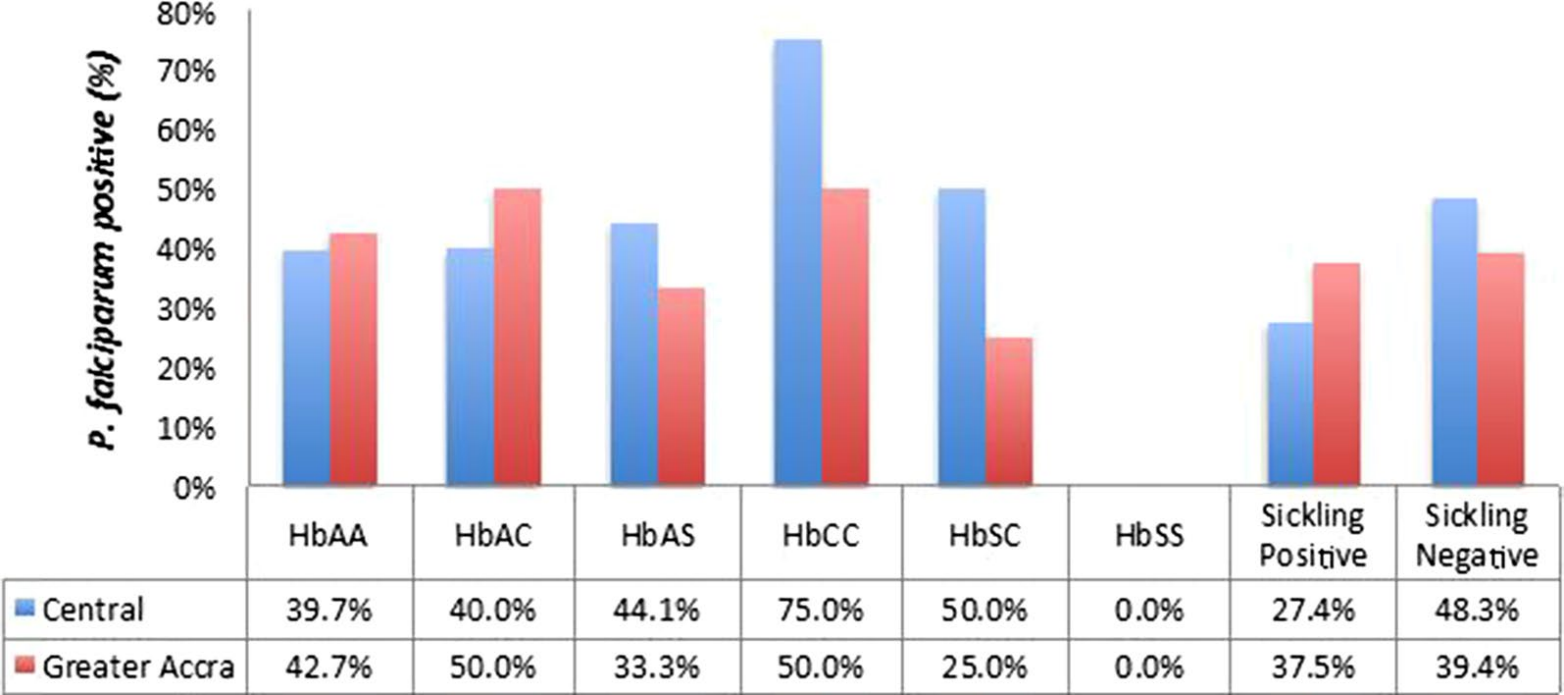
Asymptomatic *P. falciparum* carriage by the variant Hb genotypes and sickling test results. *P. falciparum* positive individuals were stratified by their Hb genotype or RBC phenotyping sickling test

Participants with HbSS were the least prevalent; only one person from the Greater Accra Region having that genotype. None from the Central Region had HbSS. HbAS was the highest abnormal haemoglobin genotype in both study populations, presenting at 16.5% and 15% in the Central and Greater Accra Regions, respectively (Fig. 3). Although there was a significant difference (Pearson Chi Square = 14.354, p < 0.0001) in the prevalence of sickling positive samples in the Central Region relative to that in the Greater Accra Region (Fig. 2a), the distribution of haemoglobin variant genotypes was similar across the two sites (Pearson Chi Square = 9.438, p = 0.150) (Fig. 3). Asymptomatic carriage of malaria parasites in participants with the most prevalent abnormal haemoglobin variants, HbAS and HbAC were similar and not different from asymptomatic carriage of malaria parasites in individuals with normal haemoglobin, HbAA (Fig. 4). Adjusting for age, sex and study area did not affect this lack of association of a particular genotype with *P. falciparum* (Additional file 1). The proportion of participants from the Central Region who were *P. falciparum* positive was higher for sickling positive participant than sickling negative participants (Fig. 4).

### Comparison between haemoglobinopathy identification techniques

**Fig. 5:**
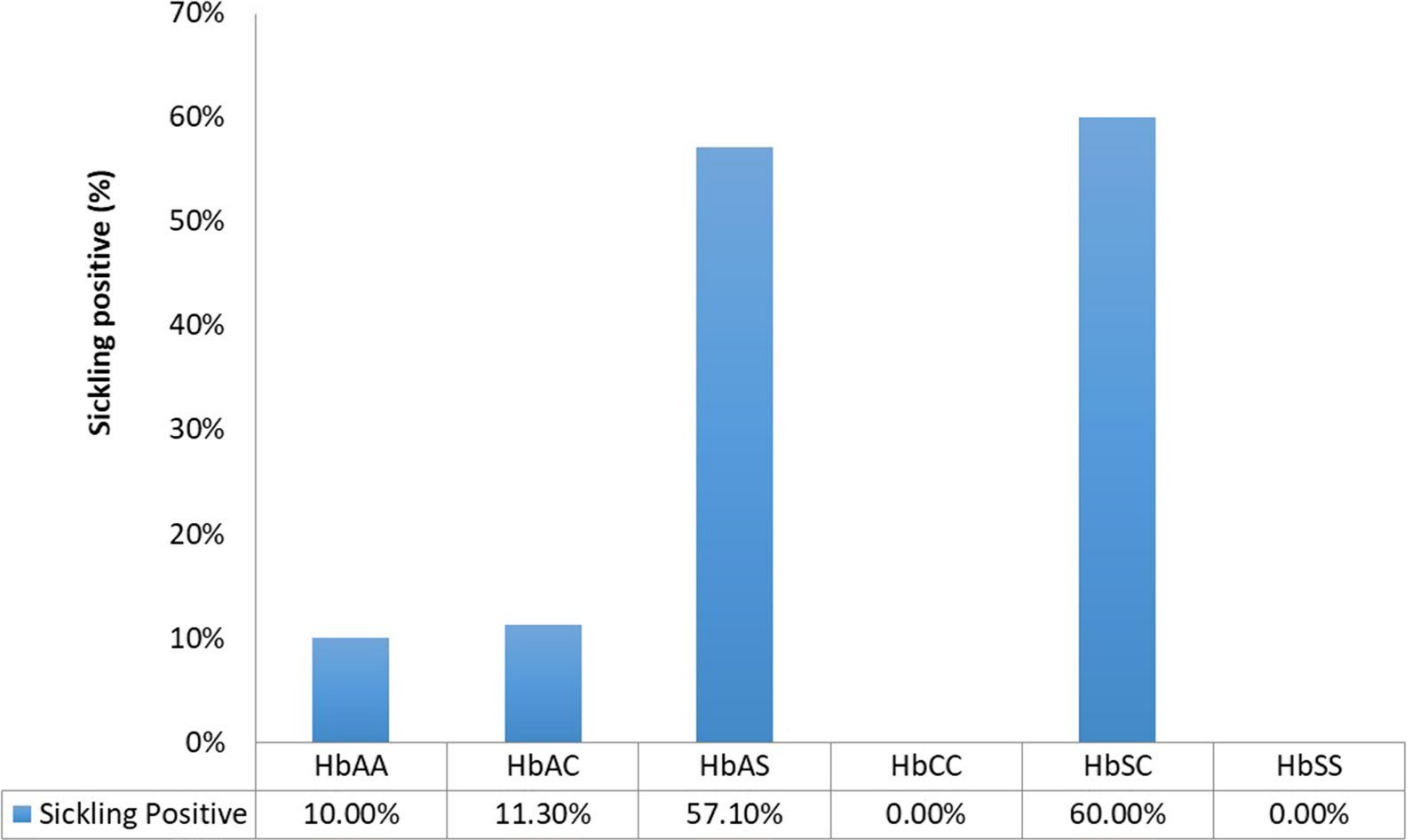
Sickling positive participants stratified by Hb genotype. The overall distribution of haemoglobin variants amongst sickling positive individuals

About 10% of HbAA and HbAC were misclassified as sickling positive, whilst about 40% of HbAS and HbSC were misclassified as sickling negative (Fig. 5). All (100%) of the HbAC and HbCC asymptomatic participants that harboured *P. falciparum* parasites were classified as sickling negative, whilst about 40% of the asymptomatic carriers with HbAS were classified as sickling negative. 5% of asymptomatic carriers with HbAA were classified as sickling positive.

### Blood group distribution in participants with variant haemoglobin genotypes

**Fig. 6:**
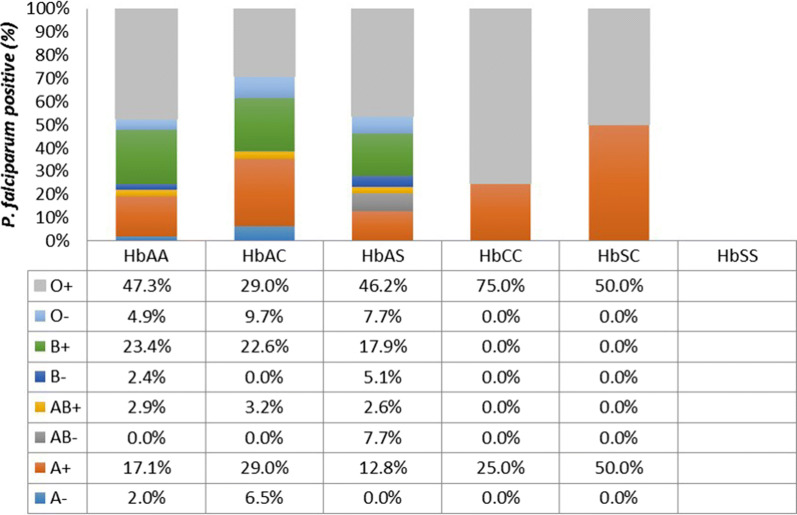
Blood group distribution of asymptomatic participants stratified by Hb genotype. The overall distribution of individuals infected with malaria parasites within a particular haemoglobin genotype stratified according to blood group antigen type

Majority of the study participants with various Hb genotypes who harbored *P. falciparum* parasites in asymptomatic infections belonged to blood group O + (Fig. 6). Blood group B + , which was the second most predominant blood group in general was present in HbAA, AC and AS only. Blood group A + was almost uniformly distributed across all the Hb variants. The only participant in the study with HbSS did not harbour any *P. falciparum* parasites as confirmed by PCR. Adjusting the data for age, sex and site did not affect the lack of association between any particular blood group type and asymptomatic *P. falciparum* carriage in a multivariate logistic regression (Additional file 1).

## Discussion

The associations between the ABO blood group antigens and haemoglobinopathies with malaria have been one of varying conclusions; especially in the case of asymptomatic parasite carriage. Whereas some studies identified blood group O as a susceptibility factor for asymptomatic *P. falciparum* infection [19], others found no association at all [20–22]. A protective role of the O over non-O blood groups have also been reported [23]. Similarly, discordant observations have been made for haemoglobinopathies and asymptomatic *P. falciparum* carriage [24, 25]. Studies that corroborate or disagree with these studies such as this study are necessary to establish facts and affirm the basis for studies into the mechanisms underlying protective associations. In this study, the distribution of ABO blood group, Haemoglobin genotypes, as well as sickling phenotypes and asymptomatic carriage of *P. falciparum* were assessed within two populations of afebrile individuals living in southern Ghana.

Asymptomatic carriage of *P. falciaprum* parasites was about 40% for both the Central and Greater Accra Region, implying a high rate of *P. falciparum* parasite carriage, even during the off-peak malaria season. Similarity of parasite prevalence in the two regions could be attributable to the fact that both regions are located within the coastal savannah zone and experience similar climatic factors [14].

The order of distribution of the blood group variants without considering rhesus factor, observed in this study (O > B>A > AB) has also been reported in previous studies in Ghana [26, 27]. There were about three times more individuals with blood group O relative to A, which is in agreement with a previous report that determined the ratio of blood group O to A to be greater than 1 in malaria endemic areas [28]. Blood group O is reported to be protective against severe forms of *P. falciparum* malaria while non-O blood groups, such as A, are associated with severe forms of the disease [2, 29].

Asymptomatic carriage of parasites was not associated with any blood group when samples were analyzed by site or as a whole, suggesting equal likelihood of each of the blood group variants being infected with *P. falciparum*. This finding is similar to previous studies done in Senegal [30], but contrary to a previous study in the Eastern Region of Ghana where blood group O and rhesus positivity were less likely to have asymptomatic *P. falciparum* infection [23]. Erythrocytes with non-O blood groups have been suggested to be more susceptible to complicated or severe forms of the disease [29]. The blood group A and B antigens have been identified to enhance the rosetting ability of *P. falciparum*, a phenomenon that contributes to the pathogenesis of severe malaria by causing occlusion of capillaries that supply oxygenated blood to vital organs such as the brain [3, 31, 32].

The most prevalent abnormal haemoglobin genotype identified was HbAS, which is generally associated with protection from *P. falciparum* infection [33] and is highly prevalent across sub-Saharan Africa where the burden of *P. falciparum* is high [34]. However, in this study, no association was identified between asymptomatic parasite carriage and any of the haemoglobin genotypes. Similar observations have been made in studies conducted in Senegal [30] and Papua New Guinea [35]. The very low frequency of individuals with HbSC and HbCC could have skewed the results obtained for asymptomatic parasite carriage in these two groups. Suggesting a larger study with more individuals with HbSC and HbSS would be needed to determine variations in asymptomatic parasites amongst individuals with full haemoglobinopathies.

Red blood cell (RBC) phenotypic assessments revealed that sickling negative individuals in the Central Region had a high rate of asymptomatic *P. falciparum* carriage than sickling positive individuals. This might be because genotypic assessment pooled individuals with various haemoglobin genotypes; each of which is not significantly associated with *P. falciparum* carriage. A significant association of sickling positive phenotype with low parasite carriage has been identified in carriers of haemoglobin S trait (AS individuals) due to the suggested role of the AS genotype in impairing *P. falciparum* infected erythrocyte cytoadherence, [7], rosette formation [36] and enhance infected erythrocyte clearance [37]. This trend of increased asymptomatic parasite carriage in sickling negative individuals was also observed in the Greater Accra Region but this was not statistically significant. This lack of association observed in the Greater Accra Region could be due a higher number of samples with normal HbAA misclassified as sickling positive and HbAC and HbAS genotypes classified as sickling negative relative to the Central Region (~ 15% in CR *vs* 20% in the GAR; Supplementary data). The misclassification obtained after haemoglobin phenotyping supports the notion that microscopy is subjective [38, 39] and that more sensitive genotyping tests including haemoglobin electrophoresis, Polymerase Chain Reaction (PCR) and high performance liquid chromatography should be preferentially used for detecting haemoglobinopathies.

## Conclusion

Asymptomatic carriage of *P. falciparum* parasites in the study population from southern Ghana was not associated with any particular blood group variant or haemoglobin genotype.

## Supplementary information

**Additional file 1.** Statistical analysis. Tables containing the exact counts of different variables determined at each site (Greater Accra and Central Region) as well as details of the Chi-Squared tests and Binary Logistic Regression analysis performed on the data.

## Data Availability

Data from which conclusion were made from this manuscript are available in the manuscript and any more details are available upon request from authors.

## Abbreviations

PCR: Polymerase Chain Reaction
Hb: Haemoglobin

## References

[1] Driss A, Hibbert JM, Wilson NO, Iqbal SA, Adamkiewicz TV, Stiles JK (2011). Genetic polymorphisms linked to susceptibility to malaria. Malar J..

[2] Panda AK, Panda SK, Sahu AN, Tripathy R, Ravindran B, Das BK (2011). Association of ABO blood group with severe falciparum malaria in adults: case control study and meta-analysis. Malar J..

[3] Uneke CJ (2007). *Plasmodium falciparum* malaria and ABO blood group: is there any relationship?. Parasitol Res.

[4] Tadesse H, Tadesse K (2013). Assessing the association of severe malaria infection and ABO blood groups in northwestern Ethiopia. J Vector Borne Dis..

[5] Piel FB, Adamkiewicz TV, Amendah D, Williams TN, Gupta S, Grosse SD (2016). Observed and expected frequencies of structural hemoglobin variants in newborn screening surveys in Africa and the Middle East: deviations from Hardy-Weinberg equilibrium. Genet Med..

[6] Williams TN, Mwangi TW, Wambua S, Alexander ND, Kortok M, Snow RW (2005). Sickle cell trait and the risk of *Plasmodium falciparum* malaria and other childhood diseases. J Infect Dis.

[7] Cholera R, Brittain NJ, Gillrie MR, Lopera-Mesa TM, Diakite SA, Arie T (2008). Impaired cytoadherence of *Plasmodium falciparum*-infected erythrocytes containing sickle hemoglobin. Proc Natl Acad Sci USA.

[8] Archer NM, Petersen N, Clark MA, Buckee CO, Childs LM, Duraisingh MT (2018). Resistance to *Plasmodium falciparum* in sickle cell trait erythrocytes is driven by oxygen-dependent growth inhibition. Proc Natl Acad Sci USA.

[9] Billo MA, Johnson ES, Doumbia SO, Poudiougou B, Sagara I, Diawara SI (2012). Sickle cell trait protects against *Plasmodium falciparum* infection. Am J Epidemiol..

[10] Agarwal A, Guindo A, Cissoko Y, Taylor JG, Coulibaly D, Kone A (2000). Hemoglobin C associated with protection from severe malaria in the Dogon of Mali, a West African population with a low prevalence of hemoglobin S. Blood.

[11] Modiano D, Luoni G, Sirima BS, Simpore J, Verra F, Konate A (2001). Haemoglobin C protects against clinical *Plasmodium falciparum* malaria. Nature.

[12] Gouagna LC, Bancone G, Yao F, Yameogo B, Dabire KR, Costantini C (2010). Genetic variation in human HBB is associated with *Plasmodium falciparum* transmission. Nat Genet.

[13] Goncalves BP, Sagara I, Coulibaly M, Wu Y, Assadou MH, Guindo A (2017). Hemoglobin variants shape the distribution of malaria parasites in human populations and their transmission potential. Sci Rep..

[14] Ayanful-Torgby R, Quashie NB, Boampong JN, Williamson KC, Amoah LE (2018). Seasonal variations in *Plasmodium falciparum* parasite prevalence assessed by varying diagnostic tests in asymptomatic children in southern Ghana. PLoS ONE.

[15] Yousuf R, Abdul Ghani SA, Abdul Khalid N (2018). Study on ABO and RhD blood grouping: comparison between conventional tile method and a new solid phase method (InTec Blood Grouping Test Kit). Malays J Pathol.

[16] Old J, Harteveld CL, Traeger-Synodinos J, Petrou M, Angastiniotis M, Galanello R. Prevention of thalassaemias and other haemoglobin disorders. Vol. 2: Laboratory Protocols. 2nd Edn. Thalassaemia International Federation, Nicosia, 2012. 24672828

[17] Adjah J, Fiadzoe B, Ayanful-Torgby R, Amoah LE (2018). Seasonal variations in *Plasmodium falciparum* genetic diversity and multiplicity of infection in asymptomatic children living in southern Ghana. BMC Infect Dis.

[18] Ayanful-Torgby R, Oppong A, Abankwa J, Acquah F, Williamson KC, Amoah LE (2016). *Plasmodium falciparum* genotype and gametocyte prevalence in children with uncomplicated malaria in coastal Ghana. Malar J..

[19] Alemu G, Mama M (2018). Asymptomatic malaria infection and associated factors among blood donors attending Arba Minch Blood Bank, Southwest Ethiopia. Ethiop J Health Sci..

[20] Bayoumi RA, Bashir AH, Abdulhadi NH (1986). Resistance to falciparum malaria among adults in central Sudan. Am J Trop Med Hyg.

[21] Montoya F, Restrepo M, Montoya AE, Rojas W (1994). Blood groups and malaria. Rev Inst Med Trop Dao Paulo..

[22] Degarege A, Gebrezgi MT, Beck-Sague CM, Wahlgren M, de Mattos LC, Madhivanan P (2019). Effect of ABO blood group on asymptomatic, uncomplicated and placental *Plasmodium falciparum* infection: systematic review and meta-analysis. BMC Infect Dis.

[23] Ofosu DN, Dotsey C, Debrekyei YM (2017). Association of asymptomatic malaria and ABO blood group among donors attending Asamankese Government Hospital. Int J Sci Res..

[24] Dongang NRR, Ngono NRA, Singh V, Koanga MML, Ngonde EMC, Moelle SA (2018). Role of genetic factors and ethnicity on the multiplicity of *Plasmodium falciparum* infection in children with asymptomatic malaria in Yaounde, Cameroon. Heliyon..

[25] Gong L, Maiteki-Sebuguzi C, Rosenthal PJ, Hubbard AE, Drakeley CJ, Dorsey G (2012). Evidence for both innate and acquired mechanisms of protection from *Plasmodium falciparum* in children with sickle cell trait. Blood.

[26] Kretchy J, Doku G, Annor R, Addy B, Asante RJSJoAMS. Distribution of ABO blood group/Rhesus factor in the Eastern Region of Ghana, towards effective blood bank inventory. 2017;5(3):821-6.

[27] Doku GN, Agbozo WK, Annor RA, Kisseh GD, Owusu MA (2019). Frequency of ABO/Rhesus (D) blood groupings and ethnic distribution in the Greater-Accra region of Ghana, towards effective blood bank inventory. Int J Immunogenet.

[28] Cserti CM, Dzik WH (2007). The ABO blood group system and *Plasmodium falciparum* malaria. Blood.

[29] Afoakwah R, Aubyn E, Prah J, Nwaefuna EK, Boampong JN (2016). Relative susceptibilities of ABO blood groups to *Plasmodium falciparum* malaria in Ghana. Adv Hematol.

[30] Vafa M, Troye-Blomberg M, Anchang J, Garcia A, Migot-Nabias F (2008). Multiplicity of *Plasmodium falciparum* infection in asymptomatic children in Senegal: relation to transmission, age and erythrocyte variants. Malar J..

[31] Moll K, Palmkvist M, Ch’ng J, Kiwuwa MS, Wahlgren M (2015). Evasion of Immunity to *Plasmodium falciparum*: rosettes of Blood Group A Impair Recognition of PfEMP1. PLoS ONE.

[32] Jötten AM, Moll K, Wahlgren M, Wixforth A, Westerhausen C (2020). Blood group and size dependent stability of *P. falciparum* infected red blood cell aggregates in capillaries. Biomicrofluidics..

[33] Kreuels B, Kreuzberg C, Kobbe R, Ayim-Akonor M, Apiah-Thompson P, Thompson B (2010). Differing effects of HbS and HbC traits on uncomplicated falciparum malaria, anemia, and child growth. Blood.

[34] Serjeant GR (2013). The natural history of sickle cell disease. Cold Spring Harbor Perspect Med..

[35] Fowkes FJ, Michon P, Pilling L, Ripley RM, Tavul L, Imrie HJ (2008). Host erythrocyte polymorphisms and exposure to *Plasmodium falciparum* in Papua New Guinea. Malar J..

[36] Luzzatto L, Nwachuku-Jarrett ES, Reddy S (1970). Increased sickling of parasitised erythrocytes as mechanism of resistance against malaria in the sickle-cell trait. Lancet.

[37] Lang PA, Kasinathan RS, Brand VB, Duranton C, Lang C, Koka S (2009). Accelerated clearance of *Plasmodium*-infected erythrocytes in sickle cell trait and annexin-A7 deficiency. Cell Physiol Biochem.

[38] Ugah UI, Alo MN, Owolabi JO, Okata-Nwali OD, Ekejindu IM, Ibeh N (2017). Evaluation of the utility value of three diagnostic methods in the detection of malaria parasites in endemic area. Malar J..

[39] Rathi S, Rathi N (2017). Reliability of Screening Tests against HPLC for the Detection of Sickle Cell Disease in Betul District-A Hospital Based Study from Central India. Natl J Integr Res Med..

